# Use of Ladle Furnace Slag and Other Industrial By-Products to Encapsulate Chloride in Municipal Solid Waste Incineration Fly Ash

**DOI:** 10.3390/ma12060925

**Published:** 2019-03-20

**Authors:** Ying Wang, Wen Ni, Prannoy Suraneni

**Affiliations:** 1 School of Civil and Resource Engineering, University of Science and Technology Beijing, Beijing 100083, China; yxw1043@miami.edu; 2 Department of Civil, Architectural, and Environmental Engineering, University of Miami, Coral Gables, FL 33146, USA; suranenip@miami.edu

**Keywords:** MSWIFA, ladle furnace slag, Friedel’s salt, chloride encapsulation

## Abstract

Municipal solid waste incineration fly ash (MSWIFA) is a hazardous by-product of waste incineration. The objective of this research is to encapsulate the chloride in MSWIFA and to develop a utilizable construction material using MSWIFA, ground granulated blast-furnace slag (GGBFS), ladle furnace slag (LFS), and gypsum. A secondary objective of the work is to explain the hydration and encapsulation mechanisms in this material system using isothermal calorimetry (IC), X-ray diffraction (XRD), thermogravimetric analysis (TGA), scanning electron microscopy (SEM), and ion chromatography (IC). The predominant hydration products are ettringite, Friedel’s salt, and C-S-H gel, with Friedel’s salt and C-S-H dominating in systems high in LFS and ettringite and C-S-H gel dominating in systems low in LFS. The chloride encapsulation showed a strong correlation with the Friedel’s salt amount; however, some encapsulation was also likely due to physical binding in the C-S-H gel. In a system with 30% MSWIFA (by mass), the optimal amount of LFS for strength and chloride encapsulation is 20%–40% (by mass).

## 1. Introduction

Incineration is a widely used and effective technology to manage municipal solid wastes, as it reduces waste volume by more than 80% [1]. However, the incineration residue, known as municipal solid waste incineration fly ash (MSWIFA), typically contains high proportions of heavy metals and hazardous ions and therefore needs careful treatment prior to disposal to reduce potential leaching of contaminants [2]. One of the ways that such powders are treated is by solidification and stabilization (S/S) using cement, which is generally considered to be an economic treatment method [3]. Some ions in MSWIFA (such as sulfate and chloride) reduce the durability of cement-based materials [3], and the high concentration of chloride in the MSWIFA may not be completely encapsulated when using cement [3,4]. Some have suggested that MSWIFA may be used as supplementary cementitious material (SCM), as it shares compositional similarities to SCMs such as ground granulated blast-furnace slag (GGBFS) [5,6,7]. However, MSWIFA shows significantly inferior performance compared to conventional SCMs [4], as it has low reactivity, retards cement hydration, and reduces the strength of cement-based materials [8].

Due to some of the drawbacks of using cement, the use of SCMs for S/S of MSWIFA would be valuable [1,2,3]. Others have shown that SCMs such as fly ash and GGBFS may be used for S/S [9] due to the presence of silica and alumina tetrahedra [3]. In this study, we explore alternative SCMs in combination with GGBFS for S/S of MSWIFA.

Ladle furnace slag (LFS) is a byproduct of the steel-making process [10,11,12]. As a residue from a high temperature (up to 1600 °C) fusion process, LFS has both amorphous and crystalline phases containing a large proportion of calcium and aluminum [8,10]. A common crystalline phase in LFS is mayenite (C_12_A_7_) [13], which can form large amounts of Friedel’s salt in the presence of chloride [14]. Friedel’s salt belongs to a class of compounds called layered double hydroxides (LDH), which consist of positively charged layers with anion-exchange properties [6]. Friedel’s salt has a high affinity for oxyanions [15,16,17], including chloride, and can be used to remove chloride from solutions [1,9,16,17,18,19]; therefore, LFS may be a promising material to encapsulate chloride in MSWIFA.

In this work, for the first time, industry by-products are used for S/S of MSWIFA. These include GGBFS, LFS, and desulfurization gypsum. The hydration, strength, and encapsulation characteristics of MSWIFA-GGBFS-LFS-gypsum systems are explored with the objective of determining the proportions of each material for optimum compressive strength and chloride encapsulation. This work is beneficial for the greater utilization of industry byproducts and MSWI fly ash, and to ensure that the MSWIFA is less harmful to the environment.

## 2. Materials and Methods

### 2.1. Materials

The MSWIFA was collected from a municipal waste incineration plant in Beijing, China. The GGBFS and gypsum were collected from Jintaicheng Environmental Resources Co., Ltd. (Xingtai, China). The GGBFS is classified as Grade S95, which means that its 7-day and 28-day strength values should be more than 75% and 95% of a reference cement mixture, respectively (according to GB/T 18046-2008, the Chinese national standard for ground granulated blast-furnace slag used for cement and concrete) [20]. LFS was obtained from Guangxi Liuzhou Iron and Steel Group Co., Ltd. (Liuzhou, China). The chemical compositions of these raw materials were determined using an X-ray fluorescence (XRF) device (FluoroMax-4, Horiba, Kyoto, Japan) following procedures similar to ASTM C 114-18. The Blaine fineness (m^2^/kg) of the raw materials was determined using ASTM C204-18. The chemical composition and fineness values are presented in Table 1. GGBFS and LFS both have large amounts of Ca. The GGBFS has a large amount of Si and a moderate amount of Al, while the LFS has a large amount of Al but negligible Si. The compositions and our research performed using a newly developed pozzolanic test (based on heat release and calcium hydroxide consumption) show that these slags are latent hydraulic [13]. The MSWIFA has a large amount of Ca and considerable amounts of Cl, S, K, and Na. The LFS has higher amounts of Al and lower amounts of Si when compared to conventional LFS materials, because Al bars were used to extract and purify the steel during manufacture (information provided by the manufacturer).

**Figure 1:**
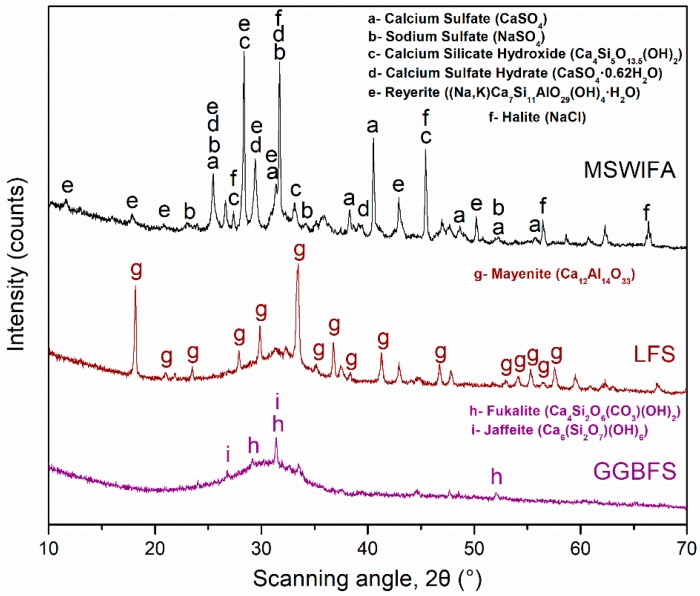
X-ray diffraction (XRD) spectra of municipal solid waste incineration fly ash (MSWIFA), ladle furnace slag (LFS), and ground granulated blast-furnace slag (GGBFS).

X-ray diffraction (XRD) (D/Max-RB diffractometer, Rigaku, Tokyo, Japan) analysis results of the raw materials are shown in Figure 1. The procedure followed for XRD is similar to that described in the experimental methods section (below). The GGBFS is largely amorphous and has minor amounts of fukalite and jaffeite. The LFS is mostly amorphous but shows large amounts of mayenite. The MSWIFA is largely crystalline and is mainly composed of calcium sulfates, sodium sulfate, calcium silicate hydroxide, halite, and reyerite. The determined crystal phases for these materials are in line with what is expected from literature [1,2,13]. 

### 2.2. Mixture Design

The seven mixtures for the four-element system prepared in this study are listed in Table 2. In all mixtures, the MSWIFA content was constant (30% of the total solid mass). In mixtures L0, L20, L40, and L60, the amount of LFS increases from 0% to 60% and the amount of GGBFS decreases from 60% to 0%. In these mixtures, the amount of gypsum is constant at 10%. In mixtures G2, G6, G10, and G14, the amount of gypsum increases from 2% to 14% and the amount of GGBFS decreases from 28% to 16%. In these mixtures, the amount of LFS is constant at 40%. It should be noted that mixtures L40 and G10 are the same. The water-to-binder ratio was maintained at 0.40, where binder is the sum of masses of MSWIFA, GGBFS, LFS, and gypsum. 

### 2.3. Experimental Methods

The materials were mechanically mixed in a mixer according to ASTM C305-14. Early-age behavior of the pastes was studied using isothermal calorimetry by monitoring the heat release for the first 168 h. Approximately 6 grams of paste was poured in a glass ampoule, which was then sealed and placed in the isothermal calorimeter (TAM Air, TA Instruments, New Castle, PA, USA), which was preconditioned at 23 ± 0.05 °C. 

The remaining paste was poured into 5 cm cubic molds. Pastes were tamped, and a vibration table with an amplitude of 0.5 mm and a frequency of 50 Hz was used for casting the mixtures. Pastes were demolded for 1 day, and the samples were cured at a humidity of 95% and at a temperature of 30 ± 2 °C. The higher temperature was used to accelerate the hydration and strength gain processes. Three samples were prepared for each test, and the curing times were 3, 28, and 90 days. The compressive strength test was performed on the samples according to ASTM C109. 

After the samples were broken, the hydration was arrested using absolute ethyl alcohol [1]. The pieces were immersed in the ethyl alcohol for 2 days, then filtered to remove the ethyl alcohol, and subsequently dried in a vacuum at 40 °C for 1 day [1]. 

Pieces were fixed on a stub, coated with carbon, and then scanning electron microscopy (SEM) (FEI FEG650, Thermo Fisher Scientific Inc., Hillsboro, OR, USA) was performed on the carbon coated pieces to evaluate the surface morphology of the hydrates using secondary electron imaging. Images were taken at 10 kV, 3000–6000×, and with a working distance of 10–12 mm. 

Other portions of the broken sample were ground and sieved through a 45-μm mesh. Three portions were obtained and used for testing XRD, thermogravimetric analysis, and leaching tests. XRD (D/Max-RB diffractometer, Rigaku, Tokyo, Japan) was performed on the powder to qualitatively determine the hydrates formed. The device was operated at 40 kV and 40 mA with a Cu Kα anode, and scanning was performed from 3° to 90° 2θ. Thermogravimetric analysis (TGA) (STA449F3, NETZSCH, Selb, Germany) was performed on the powder to quantify the amount of Friedel’s salt [21,22,23]. Approximately 30 mg of powdered sample was heated from 50 to 1300 °C at a rate of 10 °C/min in a nitrogen-purged atmosphere. 

Leaching tests were performed to determine the extent of encapsulation of the MSWIFA in the developed mixtures. The procedure was based on ASTM D8155-17. Approximately 10 g of powder samples was mixed with 100 mL of deionized water (liquid-to-solid ratio of 10) and placed in a closed conical flask. The bottles were shaken at 110 rpm on a shaking table at 23 °C for 8 h and then allowed to rest for 16 h. At the end of the extraction, the leachate was filtered with a 0.45 μm membrane filter. The ionic concentration of the leachate was detected by ion chromatography (IC) (DIONEX ICS 3000, Thermo Fisher Scientific Inc.) by injecting small amounts (1 mL) of the leachate into the injection for analysis. While the leaching concentration of chloride and other ions were measured, only chloride leaching is reported here in detail. 

The ion chromatography test was run on a MSWIFA and water mixture under the same conditions as the tested samples. Under these conditions, the MSWIFA had a very high Cl^−^ leaching concentration (20,861 mg/L (*C*0)). The Cl^−^ concentration in the leachate of each mixture (*Cx*) was determined, and the percentage encapsulation of Cl^−^ (E) is obtained using Equation (1):

(1) $$E\;=\;(1-\frac{Cx}{0.214\;\times \;C0})\times 100$$

The number 0.214 in the equation is the proportion of MSWIFA (by mass) in the paste in each group. The percentage encapsulation of Cl^−^ is assumed to be an indicator of the general encapsulation ability, as the leaching behavior of other ions was broadly similar.

## 3. Results and Discussion

### 3.1. Isothermal Calorimetry

**Figure 2:**
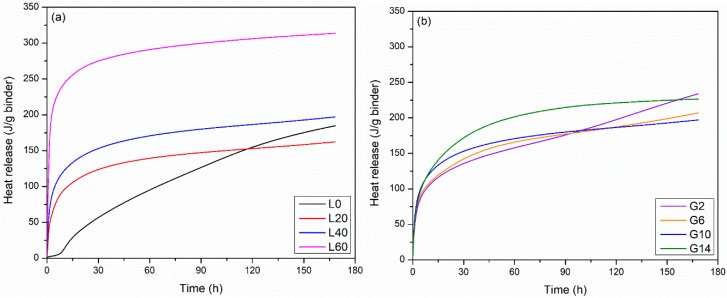
Early age heat release of (**a**) group L mixtures and (**b**) group G mixtures.

Figure 2 shows the cumulative heat release (J/g binder; referred to as heat release in the rest of the paper) for the L- and G-groups at 23 °C. The variation in the ultimate heat release for two tested replicate samples was less than 3%. According to Figure 2a, the LFS has a significant contribution of the heat release—the ultimate heat release (at 168 hours) is 185 J/g, 162 J/g, 197 J/g, and 314 J/g for mixtures L0, L20, L40, and L60, respectively. As the amount of LFS increases, the heat release increases significantly at early ages (<1 day). This is, however, not the case at later ages (7 days), as the heat release first decreases (a decrease from L0 to L20) and then increases (an increase from L20 to L60) with LFS content. This finding and the shape of the curve in general suggest that there are two processes contributing to the heat release. The initial contribution is likely because of the large amount of the C_12_A_7_ in the LFS, which can react with water and release a considerable amount of heat in the first few days of hydration [24]. In addition, the reaction of the C_12_A_7_ is also known to release aluminum ions, which can further react with the silica in GGBFS and gypsum to form C-S-H gel, react with gypsum to form ettringite, and react with Cl^−^ to form Friedel’s salt [25]. In the system L0, the reaction is likely to be between the gypsum and the GGBFS, similar to that in supersulfated slag systems. Gypsum has an effect on the heat release (Figure 2b), but, in general, all systems have a similar value of the heat release at 7 days. Greater gypsum contents likely lead to the formation of greater amounts of ettringite [14,26]. The mixture G2 shows a distinct change in the slope at approximately 4 days, which may be because of a change in the reaction mechanism when sulfate is consumed in the system and could be due to the contribution of GGBFS to the heat release [13]. 

### 3.2. Compressive Strength Evolution

**Figure 3:**
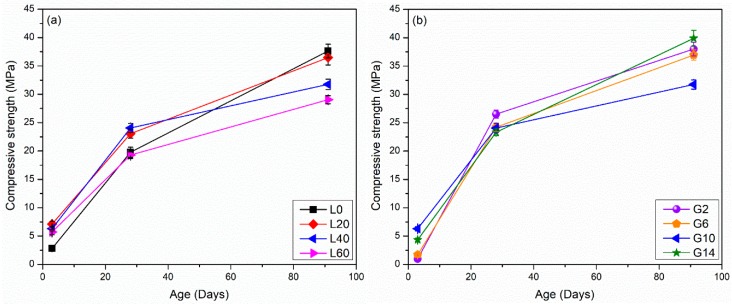
Compressive strength evolution of pastes (**a**) group L mixtures and (**b**) group G mixtures. Error bars on each side are equal to one standard deviation of the mean.

The compressive strength results of the L and G mixtures are shown in Figure 3. At each age, three replicate samples were tested and showed less than 5% coefficient of variability of the strength. In general, for all mixtures, the strengths increase over time sharply from 3 to 28 days and then somewhat more gradually from 28 to 91 days. For all mixtures, the compressive strength values at 3 days are lower than 10 MPa; however, values at 28 and 91 days are greater than 19 MPa. Mixtures with low amounts of LFS and gypsum (L0, G2, and G6) have 3-day strength lower than 3 MPa, suggesting that gypsum and LFS are crucial for the early-age strength development [25]. At ages till 28 days, mixtures with moderate amounts of LFS (L20 and L40) show the best performance in terms of strength; however, at later ages (91 days), the strength decreases as the amount of LFS increases. The L60 mixture shows a strength decrease of approximately 10 MPa (approximately 30%) compared to the L0 mixture. The following explanations for the later age strength behavior are proposed:

1. The decrease seen in mixtures with greater amounts of LFS could be due to the presence of C_12_A_7_ in the LFS, which is known to cause flash set in cementitious systems [27,28,29,30,31]. While an obvious flash setting was not observed in the mixtures here, it could potentially lead to the formation of a hard and relatively impermeable shell around LFS particles [14], which may hinder the further reaction at later ages.
2. Potential conversion of hydrates C_2_AH_8_ to C_3_AH_6_, which is known in LFS-water mixtures, may also contribute to a reduction in strength [31].
3. GGBFS is the main source of silica in the system, and later strength gain is enhanced in mixtures with greater GGBFS amounts due to the pozzolanic reaction of GGBFS to form C-S-H gel type phases, which reduces the porosity and increases the strength [10].

The effect of variations in gypsum on the compressive strength is not obvious. At 3 days, the mixtures have comparable strengths, with G10 showing the highest strength. At later ages, the mixtures G2, G4, and G14 have comparable strength, which is somewhat higher than that of G10. In theory, gypsum should help the strength gain, as it results in the formation of AFt-type phases (ettringite), which have higher strength (similar to calcium aluminate systems) [26,32,33,34,35]. However, less gypsum also implies more GGBFS, which contributes to strength gain through C-S-H gel formation. Therefore, the strength gain behavior is complex because of competing hydration and reaction mechanisms. The best 91-day compressive strength of 40 MPa is obtained for the G14 sample (30% MSWIFA, 16% GGBFS, 40% LFS, and 14% gypsum). 

In the materials studied here, heat release is not necessarily correlated with later age strength gain. As an example, L60 has a heat release that is approximately two times that of L0 at 168 h; however, these mixtures have the same strength at 28 days. This could be because of flash set of the C_12_A_7_, conversion issues in the LFS, or due to later age reaction of the GGBFS [14,31]. The heat release may not necessarily be a good predictor of the later age strength gain performance when the LFS amount changes.

### 3.3. XRD Analysis

**Figure 4:**
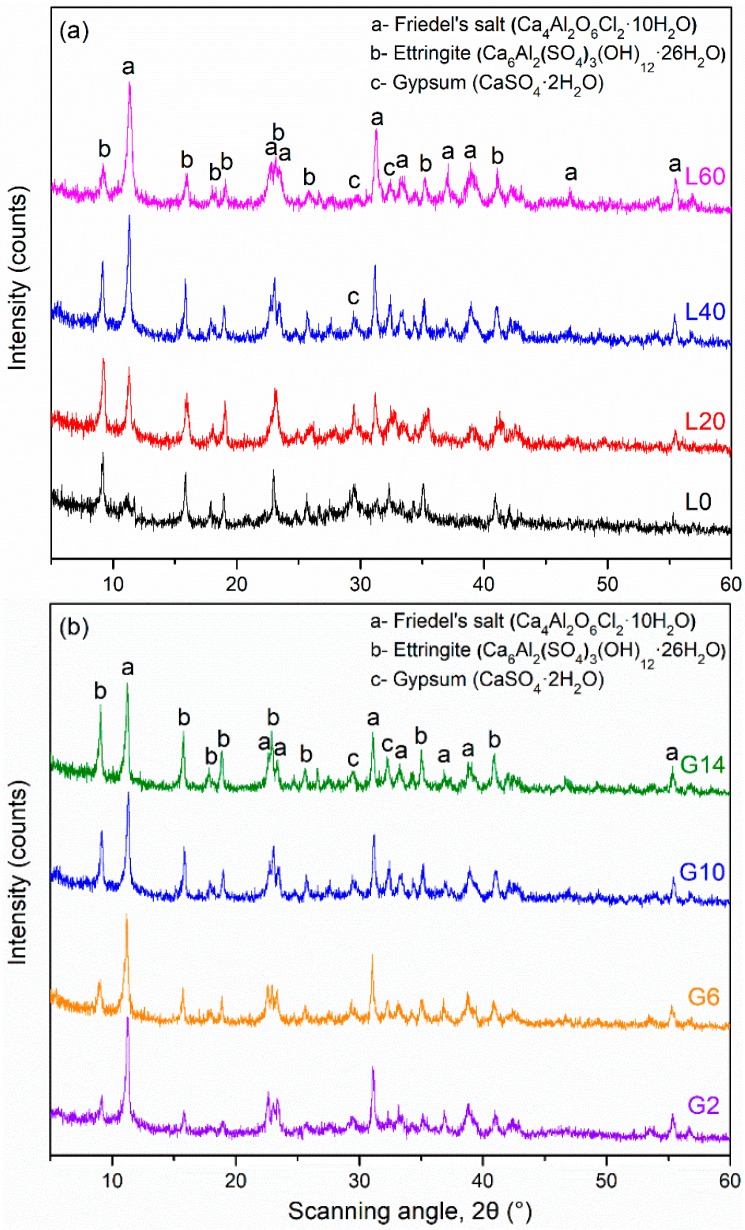
X-ray diffraction (XRD) patterns of pastes hydrated for 28 days (**a**) group L mixtures and (**b**) group G mixtures.

The XRD patterns of group L and group G pastes hydrated for 28 days are presented in Figure 4. The main hydration products are Friedel’s salt (Ca_4_Al_2_O_6_Cl_2_·10H_2_O), ettringite (Ca_6_Al_2_(SO_4_)_3_(OH)_12_·26H_2_O), and gypsum. A small amorphous hump is also seen, especially in mixtures with higher GGBFS contents. It is noted that peak heights in XRD are not a true quantitative indicator, as they depend on numerous factors (sample fineness, crystal orientation, etc.); therefore, the discussion below, which is based on peak heights, suffers from some limitations [36]. Figure 4a shows that the intensities of Friedel’s salt increase and the intensities of ettringite decrease as the amount of LFS increases. Therefore, it is tentatively concluded that the amounts of Friedel’s salt increase and the amounts of ettringite decrease as the amount of LFS increases [1]. For greater LF amounts, the aluminum coming from C_12_A_7_ may react with Cl^−^ to form Friedel’s salt before ettringite formation [22,37]. Greater LFS amounts are related to larger values of heat release, which can be seen from Figure 2. Ionic exchange and conversion mechanisms may also occur between ettringite and Friedel’s salt, which explains why the ettringite amount decreases as the Friedel’s salt amount increases [19]. Figure 4b shows that the intensity of the ettringite peaks (and presumably the amount) increases with the amount of gypsum in the system, likely due to higher amounts of sulfate coming from the gypsum. The intensity of the Friedel’s salt peaks (and presumably the amounts) decreases or remains constant as the amount of gypsum increases.

**Figure 5:**
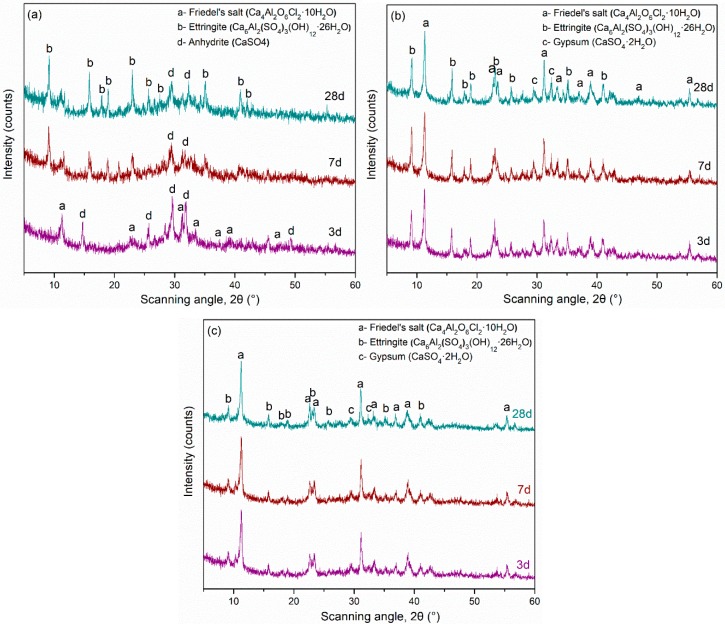
XRD patterns for pastes hydrated for 3, 7, and 28 days (**a**) L0, (**b**) L40/G10, and (**c**) G2 pastes.

Figure 5 shows the XRD patterns for L0, L4/G10, and G2 pastes hydrated for 3, 7, and 28 days. At 3 days, no ettringite peak is found in L0, while the Friedel’s salt and anhydrite peaks show the highest intensity. As hydration proceeds, Friedel’s salt and anhydrite peaks decrease in intensity, while the ettringite peaks increase in intensity. This is likely due to a reaction between GGBFS, sulfates, and Friedel’s salt to form ettringite [1,3,24]. There are no significant changes in the peak intensities of L40/G10 as hydration progresses, likely because there are adequate amounts of tetrahedrally coordinated Al and SO_4_^2−^ to maintain a steady amount of ettringite and Friedel’s salt [16]. In the mixture G2, only small amounts of ettringite and gypsum are seen, which is expected because of the low amounts of gypsum in the system [14,26]. The type and amounts of hydration products seem to be steady through the 28-day hydration process.

### 3.4. TGA Analysis

**Figure 6:**
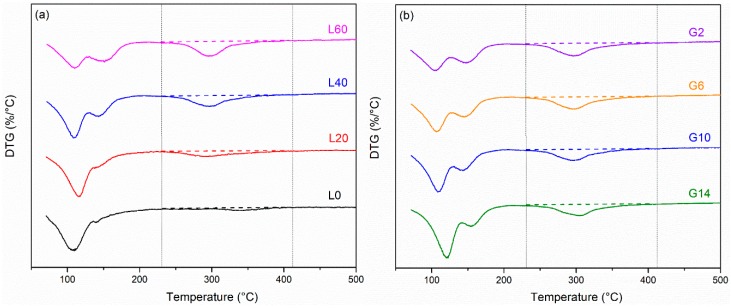
Differential thermogravimetry (DTG) showing Friedel’s salt peaks for (**a**) group L mixtures and (**b**) group G mixtures at 28 days.

Figure 6 shows the differential thermogravimetry (DTG, the first derivative of the TGA data) curves for the cement pastes of group L and G at 28 days of hydration. In the temperature range of 230–410 °C, there is no significant peak in L0, while peaks are observed in mixtures with LFS. This peak is due to the mass loss of the main layer water in Friedel’s salt, according to literature [19,22]. It is assumed here that significant water is not lost from any other phases in this temperature range, based on the shape of the peak. As shown in Figure 6a, the amount of Friedel’s salt increases as the LFS amount increases. Figure 6b shows that similar amounts of Friedel’s salt are formed in cement pastes with varying gypsum amounts. This indicates that gypsum has little influence on the formation of Friedel’s salt in pastes with adequate LFS. While the XRD does not provide quantitative results, the effects of LFS and gypsum on Friedel‘s salt amounts are similar using XRD and TGA.

**Figure 7:**
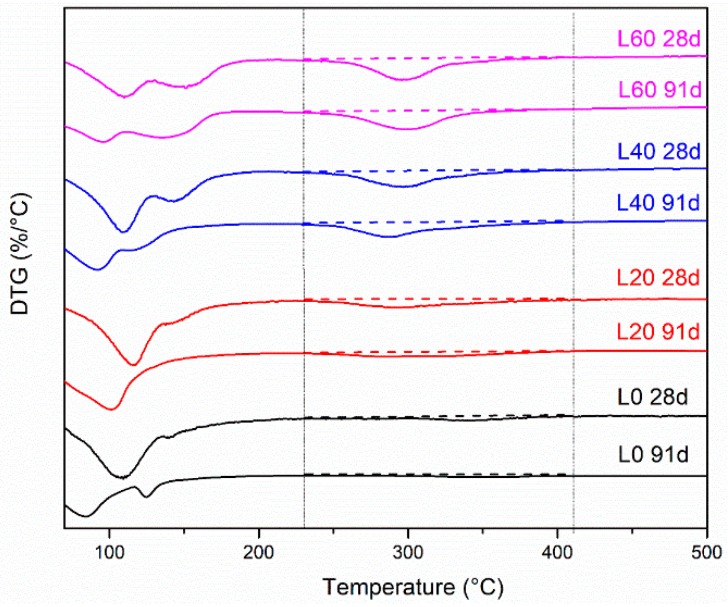
DTG showing Friedel’s salt peaks for group L mixtures as a function of time.

The mass loss for the main layer water can be determined using the area between the straight line and the peaks in the temperature range of 230–410 °C as shown in Figure 6 and Figure 7. The amounts of Friedel’s salt can then be found based on stoichiometry [19,22]. 

Some studies have shown that Friedel’s salt decomposes at later ages in some systems [1,16,38,39,40]. Figure 7 shows the variation in the DTG peaks in the L group mixtures as a function of time. No obvious decomposition of Friedel’s salt is seen in mixtures L40 and L60. This is because there is an abundant amount of Al and Cl^−^ in the system to maintain the Friedel’s salt amount [16] and keep it from decomposing. However, the amount of Friedel’s salt seems to reduce in mixtures L0 and L20, suggesting that LFS is required for the Friedel’s salt to be stable. In mixtures L0 and L20, as explained earlier, Friedel’s salt may be converted to ettringite due to reactions between with GGBFS and gypsum [1].

### 3.5. SEM Analysis

**Figure 8:**
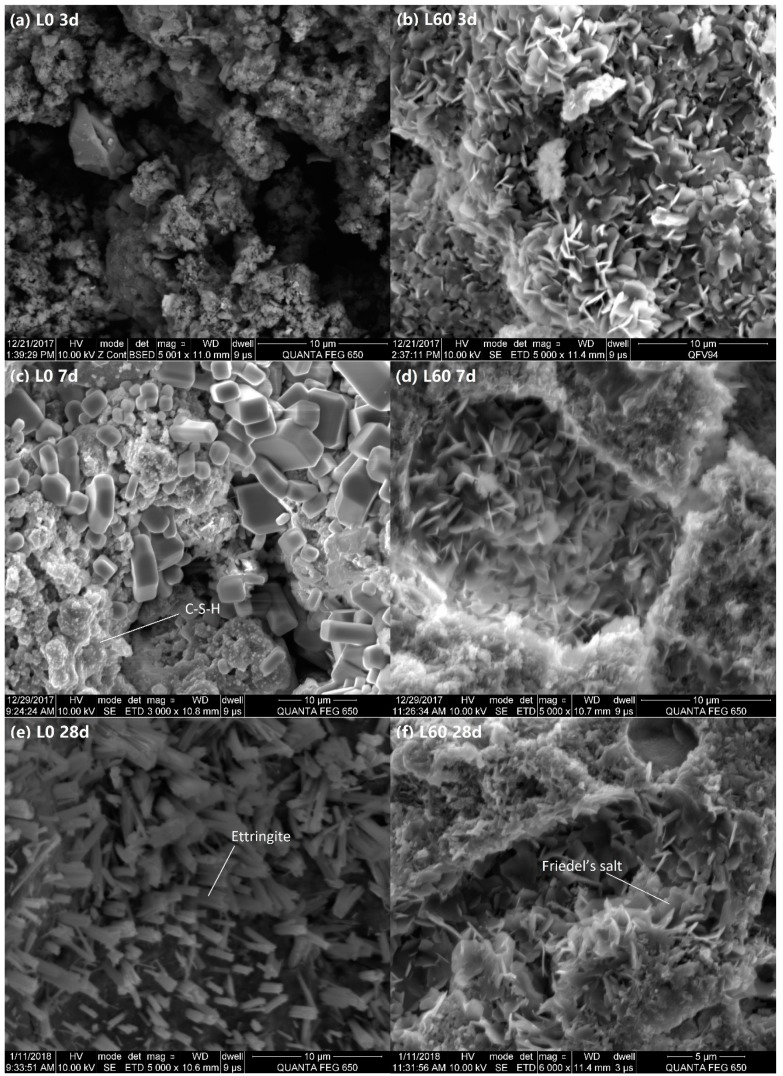
SEM images of L0 and L60 pastes at 3, 7, and 28 days. (**a**) L0 3 days; (**b**) L60 3 days; (**c**) L0 7 days; (**d**) L60 7 days; (**e**) L0 28 days; (**f**) L60 28 days.

Representative SEM images of L0 and L60 pastes hydrated at 3 days, 7 days, and 28 days are shown in Figure 8. As fracture surfaces are inherently variable, these images should only be interpreted in a qualitative manner. At 3 and 7 days, there seems to be considerable porosity in L0, based on the amount of hollow area in the micrographs. However, in L60, there is lower porosity, in addition to a significant amount of flaky hexagonal plates, which is consistent with expected Friedel’s salt morphology [39]. In both groups, at later ages, a large amount of very fine material is observed, which increases markedly from 3 days to 28 days. This material is likely to be C-S-H gel [1,25]. At 28 days, in the sample L0, a significant amount of needle-shaped ettringite [3,39] is observed. The SEM images suggest that the main hydration products of L60 are C-S-H gel and Friedel’s salt, while the main hydration products of L0 are C-S-H gel and ettringite. These results are in general agreement with TGA and XRD results discussed above. 

### 3.6. Leaching Test Results

**Figure 9:**
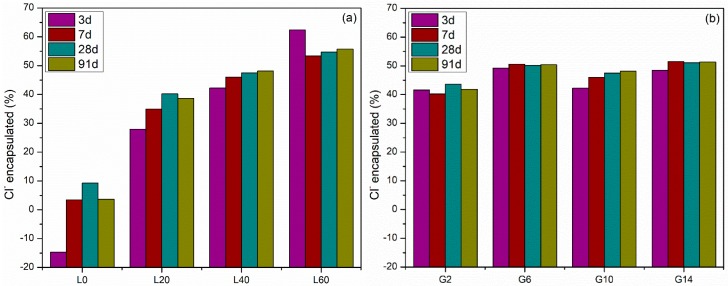
Chloride encapsulation at 3, 7, 28, and 91 days for (**a**) group L mixtures and (**b**) group G mixtures.

The percentage encapsulation of Cl^−^ of mixtures in the L and G groups hydrated for 28 days is shown in Figure 9. The tested results have less than 2% coefficient of variability in replicate testing of three samples. Figure 9a shows that as the LFS amount in the pastes increases from 0% to 60%, the chloride encapsulation increases for all ages. This increase is especially obvious in mixture L0 to L20. At 91 days, the chloride encapsulations of L0, L20, L40, and L60 are 3.7%, 38.7%, 48.1%, and 55.7%, suggesting that LFS is critical for chloride encapsulation. As waste management regulations are complex and depend on numerous factors including country, landfill location, landfill type, it is hard to determine what level of encapsulation is acceptable. For one particular case (hazardous waste acceptance at hazardous waste landfills), encapsulation percentages of greater than 45% result in leaching below acceptable limits (chloride less than 25,000 mg/kg) [41]. The 45% encapsulation corresponds roughly to an LFS content of greater than 40%. Further work is being done on waste management, but a more detailed discussion is outside the scope of this paper. Mixtures with LFS show a slight increase in the chloride encapsulation as hydration proceeds from 3 to 91 days. For the mixture without LFS (L0), there is essentially no encapsulation. Figure 9b shows that there is no obvious effect on the chloride encapsulation as the gypsum amount increases from 2% to 14%. These samples also do not show a strong effect of age on the chloride encapsulation.

### 3.7. Cl^−^ Encapsulation and Friedel’s Salt Amounts

**Figure 10:**
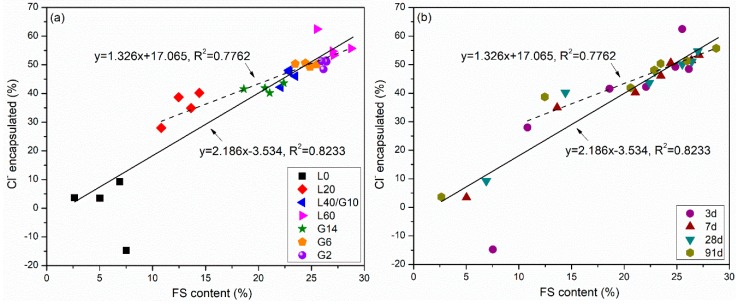
Linear relationship between Cl^−^ encapsulation and Friedel’s salt amounts showing with varying (**a**) group design and (**b**) age of hydration.

The Cl^−^ encapsulation percentage plotted as a function of Friedel’s salt amounts (which are given as a percentage of the paste mass) is shown in Figure 10. There is a linear relationship between the amount of Friedel’s salt and the chloride encapsulation in the pastes at all ages. The solid line is the regression line for all groups. The dashed line is obtained when L0 is excluded from the analysis. This may be reasonable as the L0 samples have negligible encapsulation and Friedel’s salt amounts, due to the lack of C_12_A_7_ in the system. The dashed regression line does not pass through the origin and has a positive Y-intercept at approximately 17%, indicating that there are other hydration products responsible for a portion of the chloride encapsulation. C-S-H gel, for example, is known to encapsulate chloride by physical binding [38,40,42]. When the LFS content is greater than 40%, the amount of Friedel’s salt or the chloride encapsulation do not show significant differences between groups or hydration ages. This may be because adequate amounts of Al are present in LFS, which can react with Cl^−^ at LFS contents 40% or above. The Friedel’s salt content in the group L60 is around 28%, and the Cl^−^ encapsulation percentage is around 60%, which is the best Cl^−^ encapsulation under the test conditions. Encapsulation data of chloride in MSWI fly ash and slag systems has not been studies in literature, so a direct comparison is not possible. However, similarly high Cl^−^ encapsulation rates (around 70%) are obtained using pore solution analysis on cementitious pastes with high GGBFS content (60% GGBFS, water-to-binder ratio 0.50, internal chloride 1% using NaCl) in other studies [39,43]. 

## 4. Conclusions

In this paper, isothermal calorimetry, compressive strength, X-ray diffraction (XRD), thermogravimetric analysis (TGA), scanning electron microscopy (SEM), and leaching tests were used to understand the hydration, compressive strength, and chloride encapsulation capability in municipal solid waste incineration fly ash (MSWIFA) pastes with varying amount of ground granulated blast-furnace slag (GGBFS), ladle furnace slag (LFS), and gypsum. The predominant hydration products are ettringite, Friedel’s salt, and C-S-H gel, which are principally responsible for the strength gain. In systems high in LFS, the primary hydration products are C-S-H gel and Friedel’s salt, while the main hydration products of systems low in LFS are C-S-H gel and ettringite. Similar amounts of Friedel’s salt are formed in cement pastes with LFS content higher than 40%. As the LFS content increases, the Friedel’s salt increases; however, the compressive strength decreases at later ages. Using 20% to 40% LFS and 30% MWSIFA results in mixtures with adequate strength and chloride encapsulation ability. The chloride encapsulation showed a strong correlation with the Friedel’s salt amount. Extrapolation of the relationship between chloride encapsulation and Friedel’s salt amount suggests that some encapsulation was likely due to physical binding in the C-S-H gel.

## Figures and Tables

**Table 1:** Chemical composition (mass %) and Blaine fineness (m^2^/kg) of the raw materials.

Material	CaO	SiO_2_	Al_2_O_3_	MgO	Fe_2_O_3_	Na_2_O	K_2_O	SO_3_	Cl	Blaine Fineness
GGBFS	46.54	29.78	12.18	6.00	1.22	0.45	0.41	0.36	0.02	420
LFS	55.86	0.73	36.91	3.40	1.16	0.00	0.01	0.40	0.09	400
MSWIFA	40.32	4.69	1.73	4.38	2.05	4.88	5.85	10.56	22.51	913
Gypsum	48.13	2.62	0.96	1.48	0.48	0.08	0.21	44.03	0.43	373

**Table 2:** Mixture designs of the prepared samples (in mass %).

Mixture	MSWIFA	GGBFS	LFS	Gypsum
L0	30	60	0	10
L20	30	40	20	10
L40/G10	30	20	40	10
L60	30	0	60	10
G14	30	16	40	14
G6	30	24	40	6
G2	30	28	40	2
